# Electroencephalography as a tool for evidence-based diagnosis and improved outcomes in children with epilepsy in a resource-poor setting

**DOI:** 10.11604/pamj.2015.22.328.7065

**Published:** 2015-12-03

**Authors:** Ike Oluwa Abiola Lagunju, Alexander Opebiyi Oyinlade, Omolola Mojisola Atalabi, Godwin Ogbole, Olushola Tedimola, Abimbola Famosaya, Adesola Ogunniyi, Ayotunde Oluremi Ogunseyinde, Ann Ragin

**Affiliations:** 1 Department of Paediatrics, College of Medicine, University of Ibadan/University College Hospital, Ibadan, Nigeria; 2 Department of Radiology, College of Medicine, University of Ibadan/University College Hospital, Ibadan, Nigeria; 3 Department of Medicine, College of Medicine, University of Ibadan/University College Hospital, Ibadan; 4 Department of Radiology, Northwestern University, Chicago, Illinois, USA

**Keywords:** Electroencephalography, EEG, epilepsy, childhood, diagnosis, outcome and resource-poor countries

## Abstract

**Introduction:**

Electroencephalography (EEG) remains the most important investigative modality in the diagnostic evaluation of individuals with epilepsy. Children living with epilepsy in the developing world are faced with challenges of lack of access to appropriate diagnostic evaluation and a high risk of misdiagnosis and inappropriate therapy. We appraised EEG studies in a cohort of Nigerian children with epilepsy seen in a tertiary center in order to evaluate access to and the impact of EEG in the diagnostic evaluation of the cases.

**Methods:**

Inter-ictal EEG was requested in all cases of pediatric epilepsy seen at the pediatric neurology clinic of the University College Hospital, Ibadan, Nigeria over a period of 18 months. Clinical diagnosis without EEG evaluation was compared with the final diagnosis post- EEG evaluation.

**Results:**

A total of 329 EEGs were recorded in 329 children, aged 3months to 16 years, median 61.0 months. Clinical evaluation pre-EEG classified 69.3% of the epilepsies as generalized. The a posteriori EEG evaluations showed a considerably higher proportion of localization-related epilepsies (33.6%). The final evaluation post EEG showed a 21% reduction in the proportion of cases labeled as generalized epilepsy and a 55% increase in cases of localization-related epilepsy(p<0.001).

**Conclusion:**

Here we show that there is a high risk of misdiagnosis and therefore the use of inappropriate therapies in children with epilepsy in the absence of EEG evaluation. The implications of our findings in the resource-poor country scenario are key for reducing the burden of care and cost of epilepsy treatment on both the caregivers and the already overloaded tertiary care services.

## Introduction

Electroencephalography (EEG) remains the most important investigative modality in the diagnostic evaluation individuals with epilepsy [1]. Although the diagnosis of epilepsy is clinical, EEG helps to establish the diagnosis of epilepsy, distinguish epileptic seizures from other non-epileptic events, determine the site of seizure origin and the classification of epilepsy and epilepsy syndromes [1, 2]. It helps in characterizing seizures for the purpose of treatment and it is also quite useful in prognosticating. Conventionally, epilepsy is divided into 2 broad classes; generalized and focal epilepsies and EEG has been shown to be valuable in making this distinction [3]. Misinterpretation of EEG abnormalities and lack of access to EEG evaluation are known to result in misclassification and misdiagnosis of epilepsy by physicians and prescription of inappropriate anti-epileptic drugs (AEDs) [4, 5]. Although routine inter-ictal EEG is more readily performed, continuous EEG has been shown to increase the chances of identifying epileptiform discharges in individuals with epilepsy. It is more likely to capture a seizure, capture a subclinical seizure and exclude a seizure in the patient who presents with a concerning event than routine EEG [6].

The duration of EEG recording varies among laboratories and several recommendations have been published. Craciun and colleagues in a review of 1,005 EEG recordings provided evidence for recommending at least 20 minutes recording duration for standard awake EEG and 30 minutes for sleep EEG in patients with epilepsy-related indications [7]. Electroencephalographic findings have also been reported to be useful in prognosticating in children with newly-diagnosed epilepsy and genetic generalized epilepsies [8, 9]. There is a huge burden of epilepsy in the developing countries of the world, with about 10 million persons living with epilepsy in Africa [10]. Epilepsy care in many resource-poor developing countries faces multiple challenges, which include inadequatetrained personnel, limitedaccess to EEG and other diagnostic tools, as well as lack of anti-epileptic drugs [11]. Without concomitant EEG evaluation in individuals with epilepsy, there is a risk of misclassification, wrong diagnosis and inappropriate treatment of epilepsy. The present study was undertaken to evaluate the pattern of EEG abnormalities in Nigerian children with epilepsy and to correlate seizure semiology and clinical diagnosis with EEG findings and final diagnosis.

## Methods

**Ethical approval:** Ethical approval was given by the internationally-recognized University of Ibadan/University College Hospital Ethics Research Committee (approval number UI/EC/12/0241).

**Patient recruitment and assessment:** All new cases referred to the Paediatric Neurology clinic of the UCH, Ibadan over a period of 18months, were evaluated by the Paediatric Neurologist. Electroencephalography (EEG) was requested in each child with a clinical diagnosis of epilepsy. The EEG was recorded by a trained EEG technologist using a Neurotravel 24D 32-channed EEG machine. The recordings were obtained by placing electrodes on the scalp with a conductive gel. Prior to electrode placement, the scalp area was prepared by light abrasion to reduce impedance due to dead skin cells. Electrodes were applied on the scalp using the 10-20 system [12] and recordings were acquired in the 5 standard montages-monopolar, bifronto-central, transverse, bifronto-temporal and bitemporo-occipital montages. Parents were advised to deprive the child of some sleep the night preceding the EEG examination in order to acquire a sleep EEG without the use of sedatives. Activation procedures employed in each recording session were hyperventilation (in children older than 2 years), photic stimulation, eye closure and mental activity. Each subject was required to have both a sleep and an awake record and each recording lasted 30 minutes. Each EEG record was evaluated first by the EEG technologist and later analyzed by the paediatric neurologist who has had a formal training in electrophysiology. Each record was analyzed assessing the following parameters in the background; rhythm, symmetry and wave pattern. The entire record was thoroughly reviewed for the presence of slow waves, spikes, sharp waves and spike-wave complexes, artifacts and any physiological waves. The distribution of anyabnormal rhythms and epileptiform discharges were noted and recorded when present. A final conclusion was made on each EEG recording and each record was classified as either normal or abnormal. Abnormal records in keeping with epilepsy were further classified as focal or generalized epilepsy or epilepsy syndromes based on the features identified. Final diagnosis after EEG evaluation was correlated with the initial assessment and seizure classification based on clinical evaluation, pre-EEG evaluation.

**Data entry and analysis:** Data were entered into a microcomputer and results analyzed using the statistical package SPSS for windows version 21.0.

## Results

There were a total of 329 EEG recordings from 329 children; 197 males and 132 females during the study period. Their ages ranged from 3 months to 16 years with a median of 61.0 months. Two hundred and twenty one(67.2%) children had their EEGs recorded in the awake and sleep states while 85(25.8%) and 23(7.0%) children had only awake and sleep recordings respectively. Table 1 shows the seizure types based on the clinical diagnosis of epilepsy in the 329 children. Abnormalities in the background rhythm were noted in 108 (34.7%) cases and Table 2 shows the pattern of background abnormalities.

**Table 1:** Pattern of seizures and clinical diagnosis of epilepsy in the 329 children with epilepsy

Epilepsy type	Number of cases	%
Generalized epilepsy		
Generalized tonic clonic	161	48.9
Childhood absence epilepsy	29	8.8
Atonic seizures	20	6.1
Myoclonic epilepsy	18	5.5
Partial epilepsy		
Simple partial seizures	30	9.1
Complex partial seizures	24	7.3
Partial with secondary generalization	13	3.9
Epilepsy syndromes		
West syndrome	16	4.9
Rolandic epilepsy	14	4.3
Lennox Gastaut syndrome	2	0.6
Dravet syndrome	2	0.6
**TOTAL**	329	100.0

**Table 2:** Pattern of background abnormalities in 108 children with epilepsy

Type of background abnormality	Number of cases	%
Continuous generalized slow wave activity	75	69.4
Diffuse slow wave activity with a poorly organized background	14	13.0
Asymmetry	11	10.2
Intermittent regional slow wave activity	6	5.6
Beta waves	2	1.8
**TOTAL**	108	100.0

Three hundred and ten children (94.2%) had epileptiform discharges while the remaining 19(5.8%) had normal EEG recordings. Epileptiform spikes were present in 269 (86.8%) of the EEG recordings. The spikes had a generalized distribution in 155 (57.6%), partial distribution in 67(24.9%) and partial with secondary generalization in 47 (17.5%). In children with partial-onset seizures, the left cerebral hemisphere (55.3%) was more frequently involved than the right cerebral hemisphere (44.7%). Epileptiform sharp waves were seen in 257 (78.1%) and spike-wave complexes in 44 (13.4%) of the 329 children with newly-diagnosed epilepsy. Hypsarrythmias was seen in 19 (5.8%) cases and 10 of them showed associated electrodecremental events. Spike-wave complexes were seen in 29 (8.8%) of the EEG recordings and 21 (6.4%) showed polyspikes.Table 3 shows the final diagnosis after EEG evaluation in the 329 subjects.

**Table 3:** Final seizure classification post-EEG evaluation in 310 children with EEG abnormalities

Type	Number of cases	%
Generalised epilepsy	151	48.7
Partial epilepsy	57	18.4
Partial epilepsy with secondary generalisation	47	15.2
Absence epilepsy	25	8.1
West syndrome	16	5.2
Rolandic epilepsy	10	3.2
Lennox Gastaut syndrome	2	0.6
Dravet syndrome	2	0.6
**Total**	310	100.0

Final diagnosis post-EEG evaluation showed a relatively higher proportion of localization-related epilepsy and a lower proportion of generalized epilepsy compared with the first assessment made on clinical evaluation without EEG. The final evaluation showed a 21% reduction in the proportion of cases of generalized epilepsy and a 55% increase in patients with localization-related epilepsy. McNemar test comparing diagnosis on clinical evaluation alone with final diagnosis post-EEG showed a statistically significant difference (p < 0.001). In addition, EEG significantly enhanced diagnosis in children with localization-related epilepsy compared with generalized epilepsy (p < 0.001, OR 14.84, 95% CI 7.054,31.229) (Table 4). Rolandic epilepsy was confirmed in 10 (71.4%) of the 14 suspected cases following EEG. All the 16 cases of infantile spasms identified at clinical evaluation had features consistent with a diagnosis of West syndrome on EEG evaluation.

**Table 4:** Relationship between seizure type and effect of EEG evaluation on final diagnosis

Type of Epilepsy	EEG improved diagnosis		p-value
	Yes n (%)	No n (%)	
Localisation-related	42 (40.4)	62 (59.6)	< 0.001
Generalised	11 (4.8)	214 (95.2)	
OR = 14.84			

## Discussion

EEG remains an indispensable tool in the evaluation of individuals with epilepsy. Unfortunately, in many resource-poor developing countries of the World where access to EEG is greatly limited, diagnosis relies heavily on clinical evaluation and affected individuals may have their disease wrongly classified [13, 14]. The implication of this include the prescription of inappropriate therapies, with regards to the selection of anti-epileptic drugs and greater morbidity from poor seizure control and an increased risk of mortality from poorly-treated epilepsy [15]. Our study shows a remarkably satisfactory access to EEG evaluation as all the children who were requested to have EEG were able to do so. This observation is likely to be attributed to the fact that the study was carried out in a major tertiary centre of the large urban city of Ibadan. Moreover, the group of patients seen in such a facility would be those who can access care by making the required payments in a system where healthcare is largely funded out-of-pocket. The situation is unlikely to be so in the rural areas away from the densely populated city of Ibadan where access to healthcare facilities is known to be grossly inadequate, with the myriad of challenges posed by poverty, inadequate personnel, lack of equipment and lack of reliable electricity supply.

Our study shows a high yield of electroencephalographic abnormalities, 94% in the cohort studied. This is considerably higher than the 72% yield found in a population based study in Rochester, Minnesota [16]. The authors reported that young age at diagnosis of epilepsy and idiopathic etiology increased the chances of finding epileptiform abnormalities on EEG. The high yield in our study may be related to the fact that the cohort studied was mainly paediatric. Electroencephalographic features consistent with background encephalopathies were seen in about one-third of the cases. This is consistent with previous reports, which have highlighted a relatively high prevalence of symptomatic epilepsy in Nigerian children. Intracranial infections, perinatal asphyxia and head trauma are known to be major contributors to symptomatic epilepsy in Africa [17, 18].

We show a marked reduction in the proportion of cases labeled as generalized epilepsy on clinical evaluation after the benefit of EEG. Many studies from other resource poor developing countries have consistently reported generalized seizures as the predominant seizure type in epilepsy and several of these studies have reported limited access to EEG as a major limitation [19, 20]. The opportunity of carrying out EEG evaluation provided a more accurate classification of epilepsy in our cohort. We also show the presence of localization-related epilepsy in about one-third of the cases, partial seizures in 57 (17.3%) and partial seizures with secondary generalization in 47 (14.3%) and the odds of identifying localization-related epilepsy is greatly enhanced with EEG evaluation. Focal epilepsies often present with subtle manifestations that parents, guardians and caregivers often overlook. They therefore tend to focus more on the drastic manifestations of generalized seizures, which they find more frightening. In addition, young children are often unable to give a detailed description of seizure semiology. Consequently, the sensory, autonomic and subtle motor manifestations of partial seizures may be readily missed in the absence of a corresponding EEG evaluation.

Mis-diagnosis and misclassification of epilepsy have major implications worldwide. There is a high risk of the prescription of inappropriate AEDs, which may be ineffective in controlling seizures. This may lead to frustration and increased economic burden of the disease on the individual with epilepsy and the caregivers. In situations where treatment of epilepsy is started based on clinical assessment without concomitant EEG evaluation, more children will end up requiring a change in AED therapy in order to optimize seizure outcomes following a wrong initial classification. This may result in an unnecessary prolongation of the time to seizure remission, which will in turn affect morbidity and mortality on these patients.

## Conclusion

Our study provides evidence that reinforces the need for routine EEG evaluation in all children with epilepsy, even in resource-poor settings. Indeed, facilitating access to EEG services both in urban and in rural settings of resource-poor regions of the World will, in the long run, be one of the most cost-effective intervention allowing the accurate diagnosis and treatment of children with epilepsy. This approach to the management of epilepsy will therefore improve outcomes in children with epilepsy and ultimately reduce the burden of care and cost of epilepsy on both the caregivers and the already overloaded tertiary-care public health systems. We must emphasize that there is an urgent need to improve access to EEG evaluation in individuals with epilepsy in the resource-poor countries of the world where the burden of epilepsy resides in large numbers.
